# Label-Free Fluorescent Detection of Trypsin Activity Based on DNA-Stabilized Silver Nanocluster-Peptide Conjugates

**DOI:** 10.3390/s16111477

**Published:** 2016-11-09

**Authors:** Cai-Xia Zhuo, Li-Hui Wang, Jing-Jing Feng, Yao-Dong Zhang

**Affiliations:** 1 Key Laboratory of Applied Surface and Colloid Chemistry of Ministry of Education, School of Chemistry and Chemical Engineering, Shaanxi Normal University, Xi’an 710062, China; zhuocaix@163.com (C.-X.Z.); wanglihui@snnu.edu.cn (L.-H.W.); jjfeng@snnu.edu.cn (J.-J.F.); 2 Key Laboratory of Analytical Chemistry for Life Science of Shaanxi Province, School of Chemistry and Chemical Engineering, Shaanxi Normal University, Xi’an 710062, China

**Keywords:** trypsin, silver nanocluster, graphene oxide, activity, Fluorimetry

## Abstract

Trypsin is important during the regulation of pancreatic exocrine function. The detection of trypsin activity is currently limited because of the need for the substrate to be labeled with a fluorescent tag. A label-free fluorescent method has been developed to monitor trypsin activity. The designed peptide probe consists of six arginine molecules and a cysteine terminus and can be conjugated to DNA-stabilized silver nanoclusters (DNA-AgNCs) by Ag-S bonding to enhance fluorescence. The peptide probe can also be adsorbed to the surface of graphene oxide (GO), thus resulting in the fluorescence quenching of DNA-AgNCs-peptide conjugate because of Förster resonance energy transfer. Once trypsin had degraded the peptide probe into amino acid residues, the DNA-AgNCs were released from the surface of GO, and the enhanced fluorescence of DNA-AgNCs was restored. Trypsin can be determined with a linear range of 0.0–50.0 ng/mL with a concentration as low as 1 ng/mL. This label-free method is simple and sensitive and has been successfully used for the determination of trypsin in serum. The method can also be modified to detect other proteases.

## 1. Introduction

Trypsin is one of the most significant digestive protease produced in the pancreas. It cleaves proteins or peptides at the carboxyl terminal of arginine (Arg) or lysine (Lys) residues and plays an important role in the regulation of pancreatic exocrine function [1,2]. The abnormal expression of trypsin can lead to pancreatic diseases, cystic fibrosis, and cancer [3,4]. Therefore, trypsin activity detection is important. Thus far, several strategies for trypsin assay have been reported, such as radioimmunoassay [5], colorimetry [6,7], electrochemical methods [8], and fluorescence methods [9,10,11]. Among these methods, fluorescence methods have attracted significant attention because of their simplicity in situ and in real-time detection, e.g., quantum dot (QD) conjugates based on the Förster resonance energy transfer (FRET) between QD and Cy3 [10] (i.e., QD-(peptide-Cy3)_n_), the fluorescein-labeled peptide composed of six Arg residues and graphene oxide (GO) [9], and a cationic peptide substrate labeled with *p*-nitroaniline [11]. However, these methods require fluorescence labeling and require sophisticated equipment and laborious experiment procedures. Therefore, several label-free assays for trypsin activity have been developed recently [12,13]. However, simple, sensitive, and label-free assays for trypsin are still lacking [14,15].

Few silver nanoclusters, particularly DNA-stabilized silver nanoclusters (DNA-AgNCs) [16], have been successfully applied to metal ions [17], bioactive thiols detection [18], single nucleotide mutation identification [19], methylated DNA [20], homo-adenine binding molecules [21], enzyme activities assay [22,23,24] and biological imaging [25]. The thiol compounds would allow the fluorescence enhancement of DNA-AgNCs depending on the sequence of the DNA template and the micro-environmental changes of DNA-AgNCs [26]. We previously found that thiocholine enhanced the fluorescence of DNA-AgNCs and developed a simple and rapid method for the sensitive detection of acetylcholinesterase activity [27].

GO is a single-atom-thick and 2D carbon nanomaterial with a largely hydrophobic basal plane and hydrophilic edges. Nourbakhsh et al. [28] found the oxygen plasma-treated graphene becomes luminescent depending on the level of oxidation. GO is a promising nanomaterial for use in biosensors because of its unique ability of single-stranded DNA (ssDNA) adsorption and its super fluorescence quenching property [29]. Biosensors based on the π-stacking interaction between ssDNA and GO have been investigated to detect DNA [30,31], protein [32], and enzyme activities [9,33]. The fluorescently labeled peptide can be adsorbed onto GO to achieve fluorescence quenching [32]. On the basis of these properties, the methods for the determination of thrombin [6], matrix metalloproteinase-2 [34] and kinase activity [35] have been reported. However, all methods require laborious fluorescent label procedures.

We found that peptides tagged with cysteine (Cys) can enhance the fluorescence of DNA-AgNCs, and can then be adsorbed to GO, thus resulting in fluorescence quenching. On the basis of these findings, we designed a simple peptide probe for label-free assay of trypsin activity and inhibitor screening. The peptide consists of a trypsin-cleavable peptide sequence and a cysteine terminus.

## 2. Experimental Section

### 2.1. Chemicals and Materials

A stock solution of GO (0.5 mg/mL) was obtained from Nanoon Co. Ltd. (Beijing, China). The Arg_6_-Cys peptide was purchased from Biox-vision Co. Ltd. (Anhui, China) and purified with high-performance liquid chromatography. ζ potential of GO (0.25 mg/mL) in 2.0 mM PBS (pH = 7.4) was −37.23 mV. The isoelectric point (pI) of Arg_6_-Cys was calculated to be 12.4 by a calculator. The trypsin from porcine pancreas, thrombin, alkaline phosphatase (ALP), lysozyme, and Bowman Birk inhibitor (BBI) (trypsin inhibitor) were purchased from Sigma-Aldrich (Beijing, China). Oligonucleotides with polycytosine oligonucleotide (dC_12_) were synthesized and purified by Sangon Biotechnology (Shanghai, China). Silver nitrate (99.99%), sodium borohydride (NaBH_4_ powder, 98%) and other chemicals were obtained from Alfa-Aesar (Tianjing, China) and used as received. All solutions were prepared using Millipore Milli-Q water (18.2 MΩ·cm).

UV-visible spectra were obtained using a PerkinElmer Lambda 35 spectrometer. Fluorescence spectra were obtained using a Cary Eclipse fluorescence spectrophotometer (Varian, Santa Clara, CA, USA) at room temperature. Multi-function imaging photoelectron spectroscopy (XPS) measurements were conducted on an AXIS ULTRA photoelectron spectrometer (Kratos Analytical, Manchester, UK).

### 2.2. Synthesis of dC_12_-AgNCs

The dC_12_-AgNCs were synthesized according to the previously reported method with slight modifications [36]. dC_12_ was first dissolved in 2.0 mM pH 7.4 sodium phosphate buffer. AgNO_3_ was then introduced and further incubated in an ice bath for 15 min. Finally, freshly prepared NaBH_4_ (dissolved in deionized water) was added followed by vigorous stirring for 1 min. The final concentrations were 10 μM dC_12_, 60 μM AgNO_3_, and 60 μM NaBH_4_ (mole ratio for 1:6:6). After being kept in the dark at room temperature for 2 h and 4 °C overnight, the dC_12_-AgNCs was diluted with 2.0 mM pH 7.4 sodium phosphate buffer to reach the appropriate concentration for further use.

### 2.3. Optimal Concentration of the Peptide

Different amounts of a peptide stock solution (5.0 × 10^−5^ M) were added into dC_12_-AgNCs (2.0 μM) in a 500 μL phosphate buffer (2.0 mM, pH = 7.4) to vary the peptide concentrations from 0.0 nM to 300 nM, followed by the recording of fluorescence emission intensity at 635 nm. A blank cuvette containing dC_12_-AgNCs (2.0 μM) without peptide was also recorded as the control experiment.

### 2.4. Fluorescence Quenching by GO

Different amounts of a GO stock solution (0.5 mg/mL) were added into the dC_12_-AgNCs-peptide in 500 μL phosphate buffer (2.0 mM, pH = 7.4) to vary the GO concentrations from 0.0 μg/mL to 30.0 μg/mL. After incubation at room temperature for 10 min, the fluorescence spectra of the mixtures were recorded.

### 2.5. Determination of Trypsin Activity and Inhibition

First, trypsin was diluted to 0.5, 5.0, and 50 μg/mL of 2.0 mM pH 7.4 phosphate buffer. Different amounts of the diluted trypsin were then added to a series of phosphate buffer solutions (380 μL 2.0 mM, pH 7.4) containing 2.0 μL 5.0 × 10^−5^ M peptide. After enzyme digestion at 37 °C for 20 min, the mixture was heated at 90 °C for 10 min to terminate the cleavage reaction. Finally, 100 μL 10 μM dC_12_-AgNCs and 20 μL 0.5 μg/mL GO were introduced. After another incubation of 10 min at room temperature, the fluorescence spectra were measured. Moreover, to examine the specificity of this method, some interfering proteins such as thrombin (8.0 μg/mL), ALP (20.0 U/mL) and lysozyme (2.0 μg/mL) were employed as control to replace trypsin in the detection system.

The proposed method was further used to evaluate the inhibition of trypsin by BBI. Different amounts of a BBI stock solution (1 μg/mL) were added into a series of phosphate buffer solutions (380 μL 2.0 mM, pH 7.4) containing 2.0 μL 5.0 × 10^−5^ M peptide and 5.0 μg/mL trypsin. After enzyme digestion at 37 °C for 20 min, the following experiments were performed according to the aforementioned procedure. The inhibition efficiency was determined after the measurement of trypsin activity in the presence and absence of inhibitors.

### 2.6. Dynamic Detection of Trypsin

A series of phosphate buffer solutions (380 μL 2.0 mM, pH 7.4) containing 2.0 μL 5.0 × 10^−5^ M peptide and 5.0 μg/mL trypsin were incubated at 37 °C for 0, 5, 10, 15, 20, 25, 30, 35, 40 min. After different time durations, the following experiments were performed according to the aforementioned activity assay procedure.

### 2.7. Determination of Trypsin in Serum

To verify the applicability of this method for bio-samples, the human serums of three healthy volunteers were obtained from a local hospital and were used as the complex fluid and stored at −20 °C. Given that dilution is a commonly used pretreatment procedure for protein analysis in samples with high complexity, human serum was diluted 10 times before the detection of trypsin by using the proposed method with slight modifications. Phosphate buffer (380 μL 2.0 mM, pH 7.4) containing 2.0 μL 5.0 × 10^−5^ M peptide and 100 μL diluted human serum were incubated at 37 °C for 20 min. Thereafter, 100 μL 10 μM dC_12_-AgNCs and 20 μL 0.5 μg/mL GO were introduced. After another incubation of 10 min at room temperature, the fluorescence spectra were measured. For the recovery test, the known quantities of trypsin (0.75 μg/mL) were added to the human serum. After 10 dilution processes, the following experiments were performed using the same procedure.

## 3. Results and Discussion

### 3.1. Principle Design and Feasibility

**Scheme 1:**
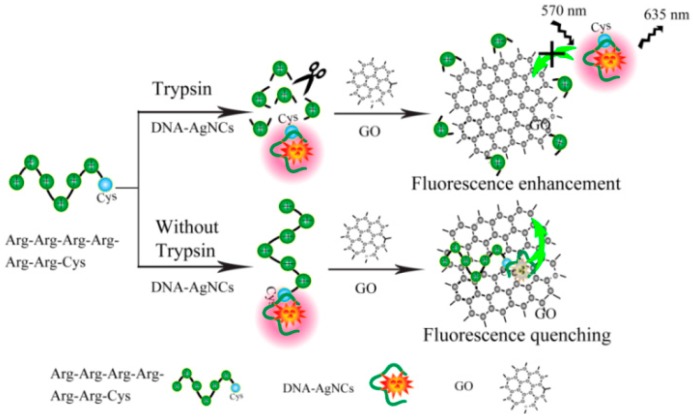
Schematic showing the principle of Arg_6_-Cys, dC_12_-AgNCs and graphene oxide (GO) for trypsin activity.

The strategy of trypsin assay is illustrated in Scheme 1. First, a trypsin-recognizing peptide with six Arg and one Cys tag (Arg_6_-Cys) was designed. The design rationale is explained as follows: (1) The peptide rapidly reacts with dC_12_-AgNCs by Ag-S bonding, thus enhancing the fluorescence of dC_12_-AgNCs; (2) After the addition of GO, the peptide was adsorbed to the surface of GO because of electrostatic interaction between the COO^−^ groups of GO and the positively charged arginine residues. Accordingly, the fluorescence of dC_12_-AgNCs was quenched because of Förster resonance energy transfer (FRET); (3) When trypsin was added, Arg_6_ was degraded into small fragments, thus resulting in the release of these fragments from the surface of GO, the disappearance of FRET, and the restoration of the fluorescence of dC_12_-AgNCs. Furthermore, the hydrolysis of the peptide catalyzed by trypsin can be inhibited by trypsin inhibitors. Therefore, a new fluorescent biosensing method for the assay of trypsin activity and its inhibitor screening can be established.

**Figure 1:**
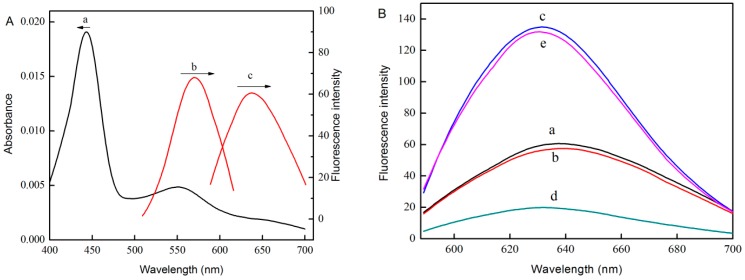
UV-Vis (curve a), excitation (curve b), and emission (curves c) spectra of dC_12_-AgNCs (**A**); Fluorescence spectrum of dC_12_-AgNCs at different conditions (**B**): (a) 2.0 μM dC_12_-AgNCs; (b) 2.0 μM dC_12_-AgNCs + 20.0 μg/mL GO; (c) 2.0 μM dC_12_-AgNCs + 200 nM peptide; (d) 2.0 μM dC_12_-AgNCs + 200 nM peptide + 20.0 μg/mL GO; (e) 2.0 μM dC_12_-AgNCs + 200 nM peptide + 200 ng/mL trypsin + 20.0 μg/mL GO.

First, we conducted the experiments to examine the feasibility of this method. The fluorescent silver nanoclusters were successfully prepared with dC_12_ as the template. The UV-vis absorption spectrum of dC_12_-AgNCs (Figure 1A, curve a) showed two peaks at 445 and 550 nm, respectively. The maximum fluorescence emission at 635 nm (Figure 1A, curve c) was obtained on excitation at 570 nm (Figure 1A, curve b). Depending on the preparation conditions, the DNA-AgNC samples can exhibit different photoluminescence characteristics. By plotting the average decay time as a function of emission wavelength emitters, a method has been developed to characterize the spectral heterogeneity and time evolution of these emissive species at any given point in time after preparation [37]. In our work, the DNA-AgNC samples were prepared and kept in the dark at room temperature for 2 h and 4 °C overnight. As shown in Figure 1B, the fluorescence of dC_12_-AgNCs (Figure 1B, curve a) is inefficiently quenched after the addition of GO (Figure 1B, curve b), thus eliminating the binding of the dC_12_-AgNCs to the GO. dC_12_-AgNCs can significantly increase because of the reaction of dC_12_-AgNCs with the peptide (Figure 1B, curve c). Moreover, the fluorescence of the dC_12_-AgNCs−peptide/GO complex is weak (Figure 1B, curve d). However, the peptide can be cleaved into small fragments in the presence of trypsin. This is a sign of peptide cleavage reaction because the same amount of inactivated trypsin could not restore the quenched fluorescence. These fragments will then be dissociated from the surface of GO, thus leading to fluorescence enhancement of dC_12_-AgNCs (Figure 1B, curve e).

### 3.2. Interaction of dC_12_-AgNCs with Peptide

**Figure 2:**
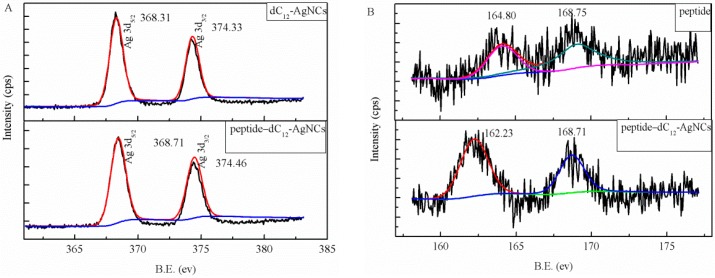
XPS spectrum of Ag 3d of dC_12_-AgNCs and dC_12_-AgNCs−peptide (**A**); XPS spectrum of S2p of peptide and dC_12_-AgNCs-peptide (**B**).

To examine the manner in which the peptide was bound to the dC_12_-Ag NCs, multi-function imaging XPS was utilized to characterize the dC_12_-AgNCs-peptide conjugate. XPS measurements showed Ag 3d_5/2_ binding energies of 368.31 and 368.71 ev for dC_12_-AgNCs and dC_12_-AgNCs-peptide conjugate (Figure 2A), respectively, corresponding to Ag (0) [38], thus indicating the existence of AgNCs. The S 2p_3/2_ peak can be deconvoluted into two different components at 164.80 eV (unbounded S)/168.75 eV (oxidation state) and 162.23 eV (Ag-S bond)/168.71 eV (oxidation state) of the peptide and dC_12_-AgNCs-peptide conjugate, respectively (Figure 2B). The presence of Ag 3d_5/2_ and S 2p_3/2_ (162.23 eV) peaks in the dC_12_-AgNCs-peptide conjugate confirm that the peptide was bound to the dC_12_-AgNCs by the Ag-S bond [39].

### 3.3. Assay of Trypsin Activity

The effects of the peptide with a Cys tag on dC_12_-AgNCs were first tested. When the peptide was added to the dC_12_-AgNCs, the fluorescence of dC_12_-AgNCs increases as a function of peptide concentration at the range of 50 nM to 250 nM and then decreases as the peptide concentration reaches 300 nM. A previous study suggested that AgNCs formed in different DNA templates can have different fluorescence response to thiol compounds [26]. The fluorescence enhancement possibly results from the charge transfer via the Ag-S bonds (from the ligands to the metal center) or from the change of the microenvironment [26,27]. The fluorescence reduction of dC_12_-AgNCs in high concentration of thiol compounds may be due to the etching of dC_12_-AgNCs induced by the large amount of thiol compounds [40]. Therefore, to balance the etching effect, the optimal concentration of the peptide substrate was chosen as 200 nM.

To investigate the fluorescence quenching effect of GO on dC_12_-AgNCs-peptide composites, various amounts of GO were employed to study the dose-dependent responses. The fluorescence is quenched dramatically after the addition of GO. The quenching efficiency reaches 85% while the concentration of GO is 20.0 μg/mL. This phenomenon is probably due to the energy transfer from the dC_12_-AgNCs unit in dC_12_-AgNCs-peptide to GO within the dC_12_-AgNCs-peptide/GO complex [32].

**Figure 3:**
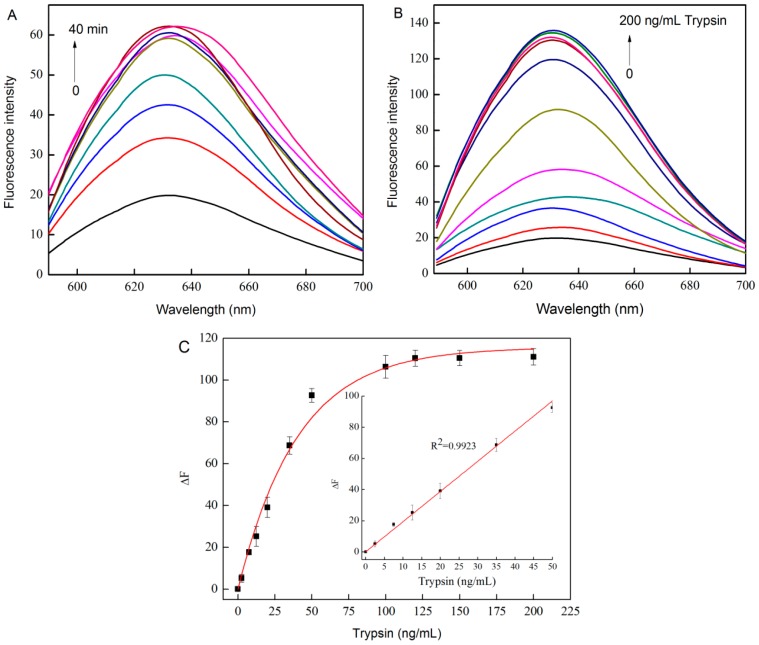
Fluorescence spectra of peptide (200 nM) in the presence of trypsin (20 ng/mL) incubated at 37 °C for different periods (**A**); Fluorescence spectra of the assay system at various concentrations of trypsin (**B**); Relationship of the fluorescence intensity changes of the assay system with trypsin concentration. Inset shows the linear response of this system to trypsin (**C**).

The application of Arg_6_-Cys peptide, dC_12_-AgNCs, and GO for trypsin activity assay was investigated. First, the solution of dC_12_-AgNCs (2.0 μM), peptide (200 nM), and GO (20.0 μg/mL) exhibited weak fluorescence. After the addition of trypsin (20 ng/mL), the fluorescence intensity at 635 nm increases with the incubation time from 0 min to 40 min (Figure 3A). The fluorescence increases quickly in the first few minutes and reaches a saturation value after 20 min, thus suggesting that the proposed method is fast and sensitive to trypsin.

Finally, the dC_12_-AgNCs-peptide composite was applied to design the FRET biosensor for monitoring trypsin activity. Under the optimum experiment conditions, various amounts of trypsin (0.0, 2.5, 7.5, 2.5, 20, 35, 50, 100, 120, 150, and 200 ng/mL) were added into peptide (200 nM) and incubated at 37 °C for 20 min. The mixtures were then heated to 90 °C for 10 min to terminate the cleavage reaction. Thereafter, dC_12_-AgNCs (2.0 μM) and GO (20.0 μg/mL) were introduced, after another incubation of 10 min, the fluorescence spectra and emission images of the mixtures were recorded. As the concentration of trypsin increased from 0.0 ng/mL to 200 ng/mL, the fluorescence intensity of dC_12_-AgNCs-peptide/GO complex was continually enhanced (Figure 3B), thus indicating a gradual cleavage of peptide. Figure 3C reveals the plot of fluorescence intensity change (ΔF) at 635 nm of dC_12_-AgNCs-peptide/GO complex vs. trypsin concentration (0.0, 2.5, 7.5, 2.5, 20, 35, 50, 100, 120, 150, and 200 ng/mL) with a saturation point near 200 ng/mL. Trypsin can be determined with a linear range of 0.0–50.0 ng/mL (inset in Figure 3C). The detection limit of trypsin was calculated as 1 ng/mL according to the rule of three times the standard deviation over the blank response, which was lower than that of some fluorescence assays reported recently [9,12,13,41,42,43]. The label-free properties of the method can offer the advantages of easy preparation of DNA-AgNCs, low cost, and operation convenience.

### 3.4. Inhibition Assay of Trypsin

**Figure 4:**
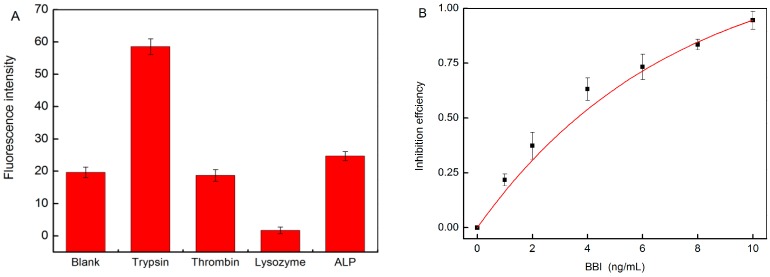
Selectivity test for trypsin (**A**). The concentrations of trypsin, alkaline phosphatase (ALP), lysozyme, thrombin are 20.0 ng/mL, 20.0 U/mL, 2.0 μg/mL, and 8.0 μg/mL, respectively; Inhibitory efficiency of BBI on the activity of trypsin (**B**). The concentration of trypsin is 20 ng/mL.

Enzymatic reactions can be retarded by its enzyme inhibitor, and some enzyme inhibitors have been utilized as drugs for diagnosis and therapy of diseases. To demonstrate that our proposed method has potential applications for trypsin inhibitor screening, BBI from soybean was selected as an example. Figure 4B depicts the plot of the inhibition efficiency versus the concentration of BBI. A higher BBI concentration in the solution leads to the slower increase in fluorescence of the dC_12_-AgNCs-peptide/GO, i.e., the inhibition efficiency of BBI on trypsin activity increases. The corresponding IC_50_ value (the concentration of the inhibitor that leads to 50% inhibition of the enzyme activity) of BBI toward trypsin was estimated to be 3.6 ng∙mL^−1^. The IC_50_ value of BBI lower than the previously reported value [9]. These results demonstrate that the developed method was sensitive for screening trypsin inhibitors.

To further assess the specificity of the proposed method, we performed a control experiment with ALP, lysozyme, and thrombin for testing. The experimental results are shown in Figure 4A. The high concentration of ALP, lysozyme, and thrombin cannot cause the observable fluorescence recovery of the dC_12_-AgNCs-peptide/GO complex at the same conditions. The etching effect of dC_12_-AgNCs [40,44] induced by large amount of lysozyme might produce a large decrease in signal. The different fluorescence patterns (turn-on or turn-off) of AgNCs in responses of thiol compounds have been reported [26,27,45]. One molecule of lysozyme (PDB 4AGA) contains 8 cysteine residues, moreover, the cysteine 6 and cysteine 127 individually locates at the terminus of lysozyme and forms disulfide bond Cys 6-Cys 127 [46]. Chymotrypsin can be selectively detected because of the different pattern of fluorescence response. This result demonstrates that the proposed method possesses high specificity for trypsin, thus indicating its applicability for bio-samples.

### 3.5. Detection of Trypsin in Human Serum

Human serum was diluted 10 times before the detection of trypsin by using the proposed method with slight modifications. Phosphate buffer (380 μL 2.0 mM, pH 7.4) containing 2.0 μL 5.0 × 10^−5^ M peptide and 100 μL diluted human serum were incubated at 37 °C for 20 min. Thereafter, 100 μL 10 μM dC_12_-AgNCs and 20 μL 0.5 μg/mL GO were introduced. After incubation of 10 min at room temperature, the concentrations of trypsin were determined to be 3.87, 4.32, and 4.45 ng/mL. After conversion (observed values multiple diluted 50 times), we obtain trypsin concentrations in human serum of 0.19, 0.22, 0.23 μg/mL (Table 1). The values were similar to those reported in previous works (0.15–0.35 μg/mL) [3,47,48]. For further evaluation of the validity of the proposed method, known quantities of trypsin (0.75 μg/mL) were added to the human serum. After 10 dilution instances, the same procedure was used to perform the following experiments. The concentrations of trypsin were determined to be 17.1, 18.4, and 17.2 ng/mL, after conversion (observed values multiple diluted 50 times), we obtain recovery values of 0.85, 0.92, and 0.86 μg/mL, respectively (Table 1). The recovery rates were determined to be 85%–94% (Table 1). These results clearly suggest that the assay can be applied to the detection of trypsin in a real sample.

## 4. Conclusions

In conclusion, we have successfully developed a new label-free fluorescence turn-on method with the ensemble of dC_12_-AgNCs-peptide and GO for trypsin assay and inhibitor screening. This new assay method is designed by taking advantage of the interactions between dC_12_-AgNCs-peptide and GO, the fluorescence quenching ability of GO, and the hydrolysis of peptide in the presence of trypsin. Trypsin can be determined with a linear range of 0.0–50.0 ng/mL with the concentration as low as 1 ng/mL. Moreover, the ensemble of dC_12_-AgNCs-peptide and GO can be employed for the screening of trypsin inhibitors with good sensitivity and the determination of trypsin in serum. This method opens new opportunities for the design of novel sensing strategies and the expansion of sensing applications in different fields.

## Figures and Tables

**Table 1:** Analytical results of trypsin determination in human serum.

Sample	Measured (μg/mL)	Trypsin Added (μg/mL)	Recovered (μg/mL)	Recovery (%)	RSD (%), *n* = 3
1	0.19	0.75	0.85	88	8.5
2	0.22	0.75	0.92	94	10.4
3	0.23	0.75	0.86	85	7.9
